# Public health emergency response through field epidemiology training and rapid response teams – a scoping review

**DOI:** 10.1186/s12889-026-27361-w

**Published:** 2026-05-01

**Authors:** Dina Mohamed Youssef, Hussein Al-Shehabi, Smilla Johann, Jennifer Kitts, Moritz Bauer, Eloisa Montt-Maray, Charbel El Bcheraoui

**Affiliations:** 1 Private Consultant, Cairo, Egypt; 2 Evidence-Based Public Health, Centre for International Health Protection, Robert Koch Institute, Berlin, Germany

**Keywords:** Public Health Emergency Workforce, Field Epidemiology training, Rapid Response Team, Pandemic preparedness, Pandemic response

## Abstract

**Background:**

Public Health Emergency Workforce (PHEW) plays a significant role in the detection and rapid response to emerging diseases, thus helping countries manage global threats. In line with the International Health Regulations’ call for strengthening national capacities, field epidemiology training programs (FETPs) and rapid response teams (RRTs) have been developed to enhance countries’ preparedness and response capacities. This scoping review synthesizes the evidence on available FETPs and RRTs and on their effectiveness as well as the challenges they face.

**Methods:**

A scoping review was conducted using EMBASE, Ovid Medline and Scopus databases in addition to the grey literature for studies published after year 2000, in the English language. Studies were selected by two independent reviewers and data were extracted into an excel sheet. Included manuscripts were analyzed through a narrative synthesis.

**Results:**

Four thousand one hundred ten studies were identified from the three peer-reviewed databases and six articles from the grey literature. Finally, 67 studies were included in the review comprising 47 identified through our search and 20 sourced from the references. The studies on PHEW training included FETPs encompassing those with laboratory and veterinary focus, and training on rapid response. Enhancement in learning acquired, course satisfaction, application of skills in workplace and engagements in key emergency response activities were found. However, lack of funding and a standardized curriculum were still among the most common challenges facing FETPs and RRTs.

**Conclusion:**

While PHEW training including FETPs and RRTs are essential for building resilience against health threats, financial challenges, lack of standardized curricula and operating procedures hinders their effectiveness. Integrating One Health and laboratory skills into FETPs are vital, as seen during the COVID-19 pandemic response. Governments should work towards increasing funding and incentivizing graduate retention. They should also collaborate with organizations such as the International Association of National Public Health Institutes (IANPHI) and the Global Field Epidemiology Partnership (GFEP) to establish standardized curricula for FETP and RRT.

**Supplementary Information:**

The online version contains supplementary material available at 10.1186/s12889-026-27361-w.

## Introduction

### Rationale

Infectious diseases transcend borders due to increased travel and trade, creating a pressing need for agile public health responses. Outbreaks such as Ebola, Yellow Fever, Zika, and most recently, the COVID-19 pandemic, have underscored the urgency to provide competency-based training to public health emergency workforce (PHEW) to enhance preparedness and effectively manage threats [1–6]. This is further stressed by the World Health Organization (WHO) positing that a trained PHEW is one of the key components to strengthen the architecture of the health emergency prevention, preparedness, response and resilience [7]. Field epidemiology training programs (FETPs) have long been considered the cornerstone of the PHEW training. FETPs are structured, competency-based training programs that build long-term capacity in applied epidemiology, typically delivered over one to two years [8]. FETPs bridge the gap between theoretical knowledge and practical application and equip PHEW with the competencies they need to support timely investigation of disease outbreaks [9]. Complementing FETPs, are rapid response teams (RRTs). RRTs are multidisciplinary teams trained through short induction, refresher courses and exercises for rapid emergency deployment [10]. Many RRT members are FETP graduates, and the programs work synergistically to strengthen national response systems and ensure rapid mobilization when outbreaks or emergencies occur [10, 11].

The Joint External Evaluation (JEE) states, regarding workforce development, that an ‘’optimal target for surveillance is one trained field epidemiologist (or equivalent) per 200,000 population. This workforce development target provides a measurable standard for IHR (2005) human resource compliance and preparedness by countries’’ [12]. Low-income countries especially suffer from a shortage in trained PHEW [13]. According to WHO data in 2006, Sub-Saharan Africa has slightly more than 10% of the world’s population, and bears 25% of the global burden of disease [14]. This is countered only by 3% of the world’s health resources and around 1% of the world’s trained health workers [14, 15]. On the other hand, while North America makes up around 14% of the global population and accounts for only 10% of the global disease burden, it possesses 42% of the world’s health workforce and accounts for about 50% of global health expenditure [15, 16]. This shows the inequitable distribution in trained global health workforce [14], exacerbated by ongoing challenges in educating and improving performance of PHEW [17, 18]. In a recent report by WHO/AFRO, it was estimated that “less than 10% of countries in the African Region have optimal and sustainable human resources to detect and respond to public health emergencies […], especially within Rapid Response Teams” [19]. Even globally, and while RRTs offer a comprehensive multidisciplinary approach required for successful outbreak response, the COVID-19 pandemic revealed that many RRT programs are not fully functional, largely due to understaffing, lack of infrastructure and financial constraints [20]. Despite the fact that both FETP graduates and RRTs had been deployed intensely during the Covid-19 pandemic, there is a limited understanding of the existing gaps, as well as the sustainability and scalability of this training beyond their immediate response. In terms of geographical distribution, there is also little evidence on the functionality and operational readiness of RRTs. Thus, evidence-based research analyzing FETPs and RRTs training, as well as identifying existing gaps is needed to inform best approaches for capability building of an integrated, multidisciplinary and multisectoral PHEW as a strategy to increase pandemic readiness.

### Aim and objectives

The aim of this scoping review is to synthesize the evidence on the curricula, diversity, geographic distribution, involvement in emergency response, and challenges of FETPs and RRTs. We also summarize the evidence on their effectiveness including comparing FETP curricula to the needs highlighted by the COVID-19 pandemic, and identifying existing gaps and challenges that FETPs and RRTs face, which require attention to enhance pandemic readiness.

## Methods

### Eligibility criteria

The inclusion/exclusion criteria of the studies in this scoping review were based on the PCC (Population, Concept, Context) framework (See Table 1) [21].

**Table 1:** Inclusion and exclusion criteria

Category	Inclusion criteria	Exclusion criteria
Population	Public health emergency workforce (PHEW). This refers to the multidisciplinary workforce responsible for delivering essential public health functions and supporting preparedness, response, and recovery during health emergencies [7, 22].	Healthcare workers (clinicians, nurses,..etc.)
Concept	Field Epidemiology training programs and initiatives, including those incorporated into academic programs, in addition to rapid response teams training. PHEW training in response to COVID-19. Also, any articles presenting gaps or challenges in these training programs	Any article not specifying the training programs of interest (generally talking about importance of educating public health workforce but not about FETP/RRT training in particular) Articles describing response efforts to an emergency without mentioning FETPS/RRT or PHEW academic training in response to COVID-19 Other public health academic training not related to FETP/RRT or not responding to COVID-19
Context	Health emergency settings globally	Non-emergency settings, non-communicable diseases
Study design	Reviews and observational studies including cross-sectional, and qualitative research. Analytical studies such as cohort, case–control, and analytical cross-sectional studies	Conference abstracts and commentaries
Language	English language studies	Language other than English
Dates	Publication date in or after 2000	Publication date prior to 2000

### Information sources

This review was reported in line with the Preferred Reporting Items for Systematic Reviews and Meta-Analyses extension for Scoping Reviews (PRISMA-ScR) guidelines (Additional file 1) [23]. EMBASE, Ovid Medline and Scopus were searched from 2000 to 2022 to identify peer-reviewed articles, and google search engine was used to identify grey literature. The search was limited to English language literature.

### Search

The research question included four concepts of search terms that were used for the search strategy (Public health workforce, emergency, training, programs). A comprehensive list of relevant synonyms and MeSH terms was used for each term. An additional file shows the final search strategy (see Additional file 2). A reference list of the studies that were selected was also reviewed to identify and include relevant references. The final search results were exported into excel sheet for screening and duplicates were removed. Mendeley was used for referencing.

### Selection of sources of evidence

**Fig. 1:**
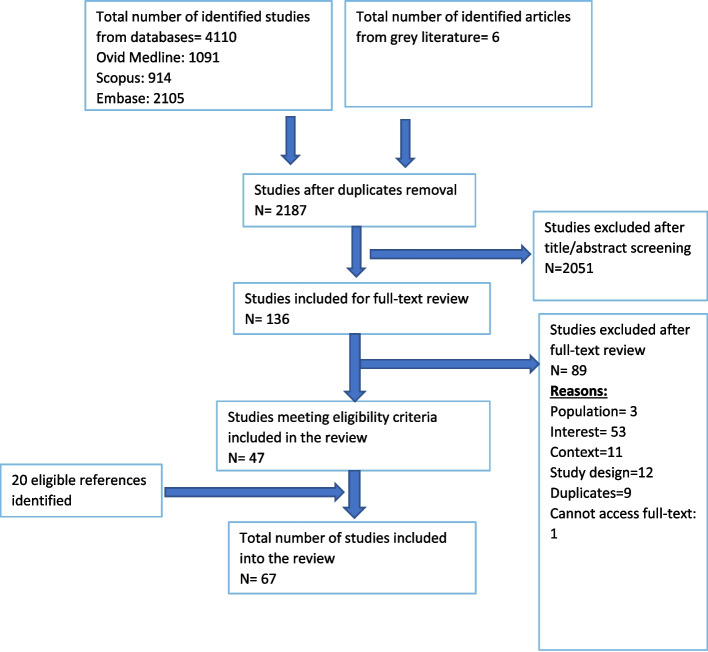
Preferred Reporting Items for Systematic Reviews and Meta-Analyses (PRISMA) flow diagram

Six reviewers (DY, MB, JK, SJ, HA, EM) contributed to the screening process. Each article was independently screened by two reviewers during both title/abstract and full-text reviewing stages. The number of articles at each stage, along with the reasons for study exclusion is presented in the PRISMA flowchart (Fig. 1). Disagreements at any stage were first discussed between reviewers; if consensus could not be reached, disagreements were resolved by a third reviewer (CEB).

### Data charting process/items

A data extraction tool was developed as an excel sheet (see Additional file 3). The following variables were extracted: Category, title of study, author, study aim, study design, study year, country, name of the training program/initiative, aim of the program, name of the organization/university/institute that developed the program, network, program/initiative description, curriculum modules or competencies or activities, suggested needs and methods, gaps/challenges, evaluation indicator, outcome of the program or evaluation, audiences, funding, criteria to recruit participants, and number of people trained.

### Synthesis of results

Included articles were analyzed using narrative synthesis. We grouped and summarized the results according to the types of training (either field epidemiology training or RRTs training). Then we presented the evidence on effectiveness of each type of training and the existing gaps.

## Results

### Study selection

Initially, 4110 peer-reviewed studies and six grey literature articles were identified. Figure 1 presents the PRISMA flow diagram for the studies included in this review. In total, 47 studies meeting eligibility were selected to be included. In addition, 20 eligible references were identified through reference harvesting. Therefore, a total of 67 studies were included in this review.

### Characteristics of sources of evidence

Study characteristics such as title, author, year, aims and objectives for each study are represented in Additional file 3. Evidence on FETPs and RRTs came from almost all regions of the world and covered training types, effectiveness, and curricula gaps and challenges.

### Types of PHEW training

Four types of PHEW training programs were identified in this scoping review: A) Field Epidemiology Training Programs (FETPs) —competency-based programs aimed at training the PHEW in field epidemiology and other public health competencies [8, 24]. —, B) Field Epidemiology Laboratory Training Programs (FELTPs) —train epidemiologists alongside laboratorians so both can gain mutual understanding of each other’s disciplines [25]. —, C) Field Epidemiology Training Programs for Veterinarians (FETPVs) —public and animal health professionals are trained and cooperate to respond to any zoonotic outbreaks [8]. —, and D) Rapid Response Teams (RRT) Training —multidisciplinary public health experts trained and ready to rapidly mobilize when there is a public health emergency [10]. These training programs are often hosted by ministries of health (MoH) in collaboration with public health institutes, academic institutions, and regional and international organizations such as the Training Programs in Epidemiology and Public Health Interventions Network (TEPHINET).

#### Field Epidemiology Training Programs (FETPs)

FETPs are mostly structured along three levels: basic or frontline (3 months) to strengthen epidemiologic capacity at the district level [26], intermediate (9 months) to train the middle level of public health professionals [27], and advanced courses (2 years). Some countries offer all three levels, while others only offer one or two depending on the context, local needs and available resources. [8] We found that most frontline FETPs were implemented in Sub-Saharan Africa, including 24 countries [28–31], mostly as a result of the 2013–2015 Ebola epidemic in West Africa [32]. Differently from other FETPs, the FETP of Papua New Guinea has a different structure consisting of five training phases over 8 months which was preferred over a traditional 2-year program in order to rapidly address capacity needs [33]. Further details about other FETPs [34–39] are available in Additional file 3.

Some FETPs were incorporated into university degrees, fellowships or a residency to build sustainable programs, such as in Jordan [40], Zimbabwe [41], Australia [42], Uganda [43], India [44], Spain [45], and the fellowship program provided by the European Center for Disease prevention and Control [46].

Regionally, a three-levels FETP was identified in five Mesoamerican countries: Costa Rica, El Salvador, Guatemala, Honduras, Nicaragua and the Dominican Republic [47]. The fellows not only responded to outbreaks, but also to natural disasters. Another regional program is the 2-year FETP of the Indian Ocean Commission countries (FETP- OI) which are Comoros, Madagascar, Mauritius, Reunion (France) and Seychelles [48].

#### Field Epidemiology and Laboratory Training Programs (FELTPs)

Field Epidemiology and Laboratory Training Programs (FELTPs) incorporate laboratory training as part of their core training curricula. FELTPs were identified mainly in Sub-Saharan Africa [49–53]. The Nigeria FELTP program is an example that aims to enhance collaboration among epidemiologists and laboratorians in the context of One Health [54, 55]. It also allows clinicians to train with veterinarians during field assignments [55]. Another example is the Namibia Field Epidemiology and Laboratory Training Program that resulted in an effective response to disease outbreaks such as Crimean-Congo hemorrhagic fever and Anthrax among hippopotamus in Bwabwata National Park, Namibia [56]. An additional file shows more details about other FELTPs and programs achievements (see Additional file 3).

#### Field Epidemiology Training Programs for Veterinarians (FETPVs)

In our review, Southeast Asia is the only identified region currently offering a regional training program for veterinary epidemiology in Bangladesh, Bhutan, Cambodia, China, Indonesia, Lao People's Democratic Republic, Malaysia, Myanmar, Nepal, Philippines, Thailand, and Viet Nam [57]. The most recently implemented FETPV was in Bangladesh in 2020 [58].

#### Rapid Response Teams (RRTs)

After the West-African Ebola outbreak in 2014/15, RRTs experienced a surge in attention as well as financial support, not only in LMICs, but also in many high-income countries like the United States, the United Kingdom (UK), Canada, and Germany [59–61]. In regions such as Africa, Asia, and the Middle East, public health organizations, e.g. the West African Health Organization, Eastern Mediterranean Public Health Network (EMPHNET), and WHO, played a significant role in facilitating the creation and training of RRTs as part of pandemic preparedness programs [62–64]. RRTs were also trained to respond to public health emergencies and conducted field investigations during the H1N1 pandemic in 2009 [65]. National RRTs operated within individual countries, coordinating emergency responses, allocating resources, and collaborating with international partners [66, 67]. Specifically during and after the West-African Ebola outbreak, RRTs received extensive training based on the WHO Ebola Virus Disease Consolidated Preparedness Checklist [68]. RRTs have also been involved in responding to other disease outbreaks such as the investigation of suspected Lassa fever cases in an Ebola-affected region in Liberia [69]. 

### PHEW training and Covid-19

The importance of FETPVs training was highlighted by the COVID-19 pandemic. In March 2020, it was agreed that a new strategic plan for the Southeast Asia FETPV needed to be developed in order to improve the epidemiological capacity of the national veterinary services [57]. Since contact tracing was crucial during COVID-19, many initiatives were established. For instance, in the USA, Butler County General Health District contracted Miami University to improve capacity building in contact tracing [70]. The Penn State College of Medicine [71] and University of California also implemented contact tracing initiatives [72]. Similarly, the Robert Koch Institute (RKI) in Germany created the Containment Scout Initiative to tackle the need for contact tracing. [73] Contact tracing also became one major surveillance activity that often actively made use of FETP graduates, such as in Yemen FETP [74]. Other FETPs contributed in the response to COVID-19 [42, 74–77].

So far there is limited evidence regarding the role and impact of RRTs during the COVID-19 pandemic. In one paper, operational challenges during Covid-19 were reported to have limited the availability and reduced the safety of RRT members during deployment [78]. Another Covid-19 specific challenge was around travel during lockdowns, resulting in delays [61]. Our review identified one paper that talked about the provincial RRT training in Papua New Guinea during Covid-19 [79].

### Effectiveness of the training programs

Many of the studies included in the review evaluated the effectiveness of FETPs/RRTS using the Kirkpatrick model for training evaluation [80], however, none of these studies followed specific criteria to evaluate FELTPs. These evaluations were based on the four levels of the model, in addition to one extra outcome found. These are learning acquired, behavior, reaction, results of training and impact on career or educational level.

#### Effectiveness of the FETP training

##### The learning acquired (knowledge, skills, or competencies)

Many evaluation studies observed improvements in competency scores [29, 31, 81–85] and in self-perceived abilities [86–88], besides increase in awareness of COVID-19 symptoms [89]. This type of evaluation helps countries to identify areas of improvement in curricula [85, 90].

##### Behavior (Application of learned skills in workplace)

Participants in many FETP training reported that skills learnt in practices positively affected their worksites [30, 82, 87] and allowed them to conduct most field epidemiology activities [91].

##### Reaction (course satisfaction)

The graduates of one FETP training reported course quantity as rather short or fair, while fieldwork duration was evaluated as right among the majority of participants. [85].

##### Results of training (in terms of targeted outcomes and engagement in key activities)

Based on FETP training, graduates were found to engage in key activities such as surveillance activities [29, 81, 84], emergency response activities [29], outbreak investigations [74, 81, 82, 85, 91, 92], risk communication efforts [74], data analysis and epidemiologic report writing [82].

##### Impact on career or educational level

FETPs were seen as beneficial towards future career advancements [82, 85, 87]. However, in some cases, the participants’ jobs are filled after they start the training, sometimes leaving fellows in lower-ranking positions after graduation [82]. This highlights the necessity to monitor career progression and recognizing FETPs as a requirement for hiring and promotion decisions.

An additional file shows further details of the indicators assessed and the evaluation outcomes (see Additional file 4).

#### Effectiveness of RRTs deployments

In the Eastern Mediterranean Region (EMR), effectiveness of RRT training in increasing skills and competency scores had been shown as well as great satisfaction with the training [62]. These RRT training have addressed issues related to the composition of an outbreak team, intersectoral coordination, surveillance systems, outbreak investigation and control measures, risk assessment, risk communication, role of the laboratory in outbreak investigation, biosafety and biosecurity, data management and report writing, and soft skills [62]. In Angola, RRTs reported using the skills and knowledge learned in a comprehensive functional exercise in Pambala [67]. The training was evaluated as valuable and that it increased the knowledge and self-perceived abilities among participants [67]. The evaluation of RRT training in Papua New Guinea during the COVID-19 pandemic showed that most participants agreed that the RRT outbreak manual was useful, and that the content was relevant to their work [79].

### Gaps and challenges

#### Gaps and challenges in FETPs/FELTPs/FETPVs

##### Challenges in curricula

Gaps related to the curriculum such as lack of health economics [91] and laboratory modules were found in EMR FETPs, with Pakistan being reported to be the only country in the EMR that included the laboratory component to FETP [88]. Also, a lack of standardized curriculum for laboratory training was found among FELTPs programs [25]. One study emphasized the importance of collaboration between laboratory scientists, geneticists, economic experts and informaticians in the field of molecular epidemiology [93]. Inadequate data analysis and computer skills training were observed in Guinea [29], Zambia [39] and India [85] FETPs. Global surveys among epidemiologists also reported lack of communication and cultural skills [94, 95]. These surveys found training gaps in emergency response surveillance, specifically in community-based and syndromic surveillance, as well as in leadership and management capacities [94] and lack of standardized curriculum for public health emergency response training [96]. Further gaps that were identified are summarized in additional file 3.

##### Job placement of graduates

Challenges of job placements and retention of graduates were reported in EMR FETP [91], Democratic Republic of the Congo (DRC) FETP [82], Yemen FETP [97] and Nigeria FELTP [54]. In some countries, for example South Africa, policies do not support paid leave, which forces professionals to either resign from their posts to enroll into the program, or decline the training offer [51]. Also, there are no clear career pathways for FELTP graduates within the departments of health, leading to many joining private organizations later on. Such situations compromise the FELTP goal of strengthening human resource capacity within the public health system [51].

##### Funding

Since funding of FELPTs in LMICs mainly depends on donors, with little or no government financial support, FELTPs in Kenya [53] and Nigeria [54], Yemen FETP [97], and the Uganda Public Health Fellowship Program [43] face budget restrictions and financial challenges [25]. Similarly, some FELTPs were stopped due to lack of funding, e.g. in Gambia, Mauritania, and Cote d’Ivoire [50]. Before revitalization of Indonesia’s FETP, weak linkages with universities and health departments compromised the financial sustainability of programs. However, revitalization of the program by incorporating it within government structures for workforce planning and budgeting are shown to be critical for building a sustainable high-quality program and maintain funding [90].

#### Gaps and Challenges of RRTs

##### Organizational challenges

Main challenges that were reported regarding establishing and managing RRTs include challenges in developing and maintaining a functional RRT roster in addition to lack of a standardized competency-based training. These challenges can lead to critical delays in outbreak response [98]. Further challenges that were reported include the composition and size of teams, development and adherence to standard operating procedures for training and deployments, ensuring rapid response times, ensuring the safety of team members in high-risk areas, addressing geographical barriers, and securing adequate funding for missions [98].

##### Infrastructure challenges

The lack of logistics hubs and adequate infrastructure within the African region was reported to be a big challenge that limits the availability of emergency supplies and stockpiles and also exacerbates response time [98]. Instead of the foreseen rapid response time of 24 to 48 h, it can take up to 20 days before countries receive much needed supplies [19].

##### Other challenges

Ongoing regional conflicts in outbreak affected areas (e.g. Ebola in North Kivu (DRC)), emergence of co-infections and outbreaks of other infectious diseases were also reported to hamper the impact of RRT response activities [99]. Further, RRTs have been reported to face challenges regarding cultural sensitivity, effective communication, as well as gaining trust of the communities [98].

## Discussion

### Summary of evidence

This review summarized the evidence on PHEW training including field epidemiology and RRTs and their curricula. It also reported on training evaluations and highlighted the existing gaps. Overall, 67 studies met the eligibility criteria and were included in the review. While both FETPs and RRTs contribute to epidemiological capacity and outbreak response, they serve distinct purposes. FETPs are comprehensive training programs aimed at building long-term epidemiological capacity, whereas RRTs are specialized teams activated for rapid response during emergencies. The most common field epidemiology training found were FETPs, while more FELTPs were found in Sub-Saharan Africa [25, 49, 50]. The deployment of FETP trainees and alumni in public health emergencies, such as the Ebola outbreak in Liberia [92] and the COVID-19 response in Yemen, Uganda, India, and Australia [42, 74–77] highlights the strength of FETPs’ experiential, on-the-job training model, which equips participants to actively contribute to both national and international response efforts. The example of Jordan FETP engagement in gathering data on refugees indicates that not only disease outbreaks but also humanitarian crises make use of FETPs fellows [85]. FETPs of the Mesoamerican countries are another good example showing the importance of FETPs fellows in responding to other crises such as natural disasters [47]. The review also found an increase in the RRTs training in both low- and high-income countries especially after the Ebola outbreak in west-Africa.

While most of the studies evaluating the training showed improvements in skills and reported high course satisfaction, some training’s gaps were identified in this review. The findings of the review emphasize the need for more laboratory training, as some countries had discontinued their FELTP programs, mainly due to financial restrictions and lack of a standardized curriculum [25, 50, 88]. This aligns with the results that only one country in the EMR, Pakistan, reported training in laboratory skills [88]. Among the included studies, few curricula included veterinary epidemiology modules. Also, only one region offering a veterinary epidemiology training program was captured in this review [57]. This reflects the need for increasing the capacity in veterinary epidemiology since previous studies have shown its benefits in response to COVID-19 [100].

Despite the importance of contact tracing and IPC skills in response to the COVID-19 pandemic, a lack of training on these topics within FETPs curricula were identified in this review [88]. This underlines the need to incorporate contact tracing training through FETPs since a previous systematic review has shown its effectiveness in reducing infectious disease cases [101]. It also highlights the need to conduct more research on the capacity of contact tracing training and whether their curricula are standardized or not. Moreover, this review showed that funding is a main challenge for all PHEW training sustainability. Although FETPs/FELTPs are prestigious training, there are still gaps in graduates’ retention in their positions after the training, leading to inadequate applicability of learned skills in their countries [54, 82, 97].

Rapid response teams are at the forefront of public health emergencies and outbreak response. However, before the West-African Ebola epidemic and more recently the Covid-19 pandemic, RRTs on the national level were often improvised, non-formalized and tended to disappear after the interventions ended [20].

### Recommendations

Given the limited representation of veterinary epidemiology training identified in this review, it is recommended that animal health perspectives be more systematically integrated into FETPs and FELTPs. Strengthening this component would support a more comprehensive approach to zoonotic disease detection, investigation, and preparedness within the broader One Health framework. To support this, the UN Food and Agriculture Organization has developed core competencies and guidelines to facilitate the developments of such training [102, 103]. Laboratory skills such as genomic sequencing and surveillance were crucial during the COVID-19 response to monitor the emergence of new variants [104, 105]. Thus, FETPs training should consider incorporating a genomic component to help epidemiologists interpret and apply genomic results. WHO has developed a “Global genomic surveillance strategy 2022–2032” [106]. One of its main objectives is rolling-out training packages in genomics and bioinformatics to improve competencies and strengthen programs for workforce development.

Since PHEW training differ between countries worldwide in terms of their curricula, governments in collaboration with organizations such as the International Association of National Public Health Institutes (IANPHI) [107] and the Global Field Epidemiology Partnership (GFEP) [108], may work toward establishing standardized curricula for FETPs and RRTs. To ensure workforce retention, MOHs may consider not only financial incentives but also broader strategies such as providing clear career pathways, more opportunities for continued training and professional development and integration of FETPs/FELTPs graduates into permeant public health roles. Since many FELTPs in low- and middle-income countries face financial challenges due to heavy reliance on external donor funding, it is recommended that governments explore more stable domestic funding mechanisms. Also, incorporating FELTPs within key MOH’s department structures for workforce and financial planning may also enhance long-term sustainability. Strengthening collaborations with universities and international partners may also help diversify funding sources. While countries may consider financial intermediary funds that support pandemic preparedness, and response, the primary focus should remain on building resilient and locally supported systems to reduce dependency on external funding sources [109].

Countries should also establish a well-functioning RRT roster and enhance the infrastructure to improve RRT management. The US Centers for Disease Control and Prevention (CDC) recommends establishing formal Rapid Response Teams (RRTs) at both national and subnational levels, integrated within public health systems. This includes defining roles, developing standard procedures, maintaining trained personnel rosters, and conducting regular training and simulations. The guidance also stresses the need for clear leadership and strong links to emergency coordination systems to ensure timely outbreak response [10].

### Strengths and limitations

To the best of our knowledge, this review is the first to synthesize the evidence on FETPs and RRTs globally. The review followed a systematic search strategy to comprehensively map the available evidence. However, as our search was limited to the English language, we could have missed topic-relevant literature published in other languages. Although a more detailed search of the grey literature was not feasible due to time restrictions, most of the grey literature found reported similar types of programs as those identified from peer-reviewed databases. Further, study selection was conducted by five independent reviewers, while other reviewer helped in resolving disagreements to decrease risk of bias in study selection. The included studies ranged in publication dates from 2000 to 2022, and more recent papers may include new PHEWs training being implemented such as contact tracing training and FETVPs.

## Conclusion

Competency-based training for the PHEW is essential in strengthening national capacity for the prevention, preparedness, response to health emergencies. Evidence from this review indicates that FETPs and RRTs improve technical knowledge, facilitate practical application of skills, and increase engagement in key outbreak response activities, highlighting their vital role in pandemic control. However, standardizing training programs, securing funding, ensuring workforce retention, integrating veterinary and laboratory components, and enhancing infrastructure are all needed to scale up these training programs. Addressing these gaps will strengthen PHEW capacity to respond to future zoonotic and infectious disease threats.

## Supplementary Information

Additional file 1: Preferred Reporting Items for Systematic reviews and Meta-Analyses extension for Scoping Reviews (PRISMA-ScR) Checklist.

Additional file 2: Search Terms of EMBASE, Ovid Medline, and Scopus.

Additional file 3: Data Extraction: The data extraction for all included studies.

Additional file 4: Evaluation results of Field Epidemiology Training Programs (FETPs): Evaluation of the effectiveness of FETPs that is presented in the studies.

## Data Availability

No datasets were generated or analysed during the current study.

## Abbreviations

PHEW: Public Health Emergency Workforce
FETPs: Field Epidemiology Training Programs
RRTs: Rapid Response Teams
JEE: Joint External Evaluation
IHR: International Health Regulations
WHO: World Health Organization
PCC: Population, Concept, Context
PRISMA-ScR: Preferred Reporting Items for Systematic Reviews and Meta-Analyses extension for Scoping Reviews
FELTPs: Field Epidemiology Laboratory Training Programs
FETPVs: Field Epidemiology Training Programs for Veterinarians
EMPHNET: Eastern Mediterranean Public Health Network
TEPHINET: Training Programs in Epidemiology and Public Health Interventions Network
MoH: Ministries of Health
FETP- OI: Indian Ocean Field Epidemiology Training Program
UK: United Kingdom
CDC: Centers for Disease Control and Prevention
DRC: Democratic Republic of the Congo
IPC: Infection Prevention and Control
EMR: Eastern Mediterranean Region
IANPHI: International Association of National Public Health Institutes
GFEP: Global Field Epidemiology Partnership

## References

[1] Nsubuga P. The Ebola outbreak in West Africa: a story of related public health challenges and a pointer to solutions to mitigate the inevitable next outbreak. Pan Afr Med J. 2014;19:1–5. 25667710 10.11604/pamj.2014.19.48.5336 PMC4315480

[2] Ario AR, Wanyenze RK, Opio A, Tusiime P, Kadobera D, Kwesiga B, et al. Strengthening global health security through Africa’s first absolute post-master’s fellowship program in field epidemiology in Uganda. Heal Secur. 2018;16:S87-97. 10.1089/hs.2018.0045 PMC13175242 30480499

[3] Olu O, Usman A, Kalambay K, Anyangwe S, Voyi K, Orach CG, et al. What should the African health workforce know about disasters? Proposed competencies for strengthening public health disaster risk management education in Africa. BMC Med Educ. 2018;18(1):1–10. 29609618 10.1186/s12909-018-1163-9 PMC5879558

[4] Haldane V, Jung AS, De Foo C, Bonk M, Jamieson M, Wu S, et al. Strengthening the basics: Public health responses to prevent the next pandemic. BMJ. 2021;375(fig 1):1–4. 10.1136/bmj-2021-067510 PMC8624065 34840134

[5] MacIntyre CR, Binkin N. In the room where it happens: the consequences of the lack of public health expertise during the COVID-19 pandemic. Glob Biosecurity. 2021;3(1):2–5.

[6] The World Bank. Cannot Wait Change - Building resilient health systems in the shadow of COVID-19. 2022. Available from: https://openknowledge.worldbank.org/server/api/core/bitstreams/a560d0b2-b2dc-5800-84a3-0ac0b29477cc/content.

[7] World Health Organization. Strengthening health emergency prevention, preparedness, response and resilience. 2023. Available from: https://cdn.who.int/media/docs/default-source/emergency-preparedness/who_hepr_wha2023-21051248b.pdf?sfvrsn=a82abdf4_3&download=true. Cited 2024 Feb 4.

[8] TEPHINET. About FETPs. Available from: https://www.tephinet.org/about/about-fetps. Cited 2023 Apr 18.

[9] Center for Deasease Control and Prevention. Field Epidemiology Training Program Development Handbook. 2006;1–107. Available from: http://www.cdc.gov/globalhealth/FETP/pdf/FETP_development_handbook_508.pdf. Cited 2023 Sep 5.

[10] Centers for Disease Control and Prevention. Establishment and Management of Public Health Rapid Response Teams for Disease Outbreaks: Covid-19 Disease Supplement. 2020. Available from: https://stacks.cdc.gov/view/cdc/125227.

[11] EMPHNET. EMPHNET: History. Available from: https://emphnet.net/en/our-story/history?utm_source=chatgpt.com. Cited 2025 Jul 3.

[12] Williams SG, Fontaine RE, Turcios Ruiz RM, Walke H, Ijaz K, Baggett HC. One field epidemiologist per 200,000 population: lessons learned from implementing a global public health workforce target. Heal Secur. 2020;18(S1):S113-8. Available from: https://www.researchgate.net/publication/338960203_One_Field_Epidemiologist_per_200000_Population_Lessons_Learned_from_Implementing_a_Global_Public_Health_Workforce_Target. Cited 2023 Mar 12. 10.1089/hs.2019.0119 PMC11361411 32004135

[13] Jones DS, Dicker RC, Fontaine RE, Boore AL, Omolo JO, Ashgar RJ, et al. Building Global Epidemiology and Response Capacity with Field Epidemiology Training Programs. Emerg Infect Dis. 2017;23(Suppl 1):S158 Available from: /pmc/articles/PMC5711325/. Cited 2023 Mar 16. 29155658 10.3201/eid2313.170509 PMC5711325

[14] Crisp LN. Global health capacity and workforce development: turning the world upside down. Infect Dis Clin North Am. 2011;25:359 Available from: /pmc/articles/PMC7127544/. Cited 2023 Mar 12. 21628051 10.1016/j.idc.2011.02.010 PMC7127544

[15] World Health Organization. Health workforce: The health workforce crisis. Available from: https://www.who.int/news-room/questions-and-answers/item/q-a-on-the-health-workforce-crisis?utm_source=chatgpt.com. Cited 2025 Jul 5.

[16] Global Health learning. Global Shortage of Health Workers and its Impact. Available from: https://www.globalhealthlearning.org/sites/default/files/page-files/Global_Shortage_of_Health_Workers.pdf. Cited 2025 Jul 5.

[17] Okoroafor SC, Asamani JA, Kabego L, Ahmat A, Nyoni J, Millogo JJS, et al. Preparing the health workforce for future public health emergencies in Africa. BMJ Glob Health. 2022;7:e008327. 35414522 10.1136/bmjgh-2021-008327 PMC9006823

[18] Beaglehole R, Dal Poz MR. Public health workforce: challenges and policy issues. Hum Resour Health. 2003;1(1):4. Available from: http://www.human-resources-health.com/content/1/1/4. Cited 2022 Aug 23. 12904251 10.1186/1478-4491-1-4 PMC179882

[19] World Health Organization. Ensuring Health Security in the African Region. 2022. Available from: https://www.afro.who.int/sites/default/files/2022-05/WHOAFROEPR_Quarterlyreport%231_WEBversion_English.pdf.

[20] Ten Bousso A. Actions for Africa to be ready for the Next Pandemic. Medicon Med Sci. 2022;3(3):12–4. Available from: https://themedicon.com/pdf/medicalsciences/MCMS-03-063.pdf.

[21] JBI. Inclusion criteria - JBI Manual for Evidence Synthesis. 2022. Available from: https://jbi-global-wiki.refined.site/space/MANUAL/4687714/11.2.4+Inclusion+criteria. Cited 2024 Jan 29.

[22] Mosam A, Fisher DA, Hunter MB, Kunjumen T, Mustafa S, Nair TS, et al. Public health and emergency workforce: a roadmap for WHO and partner contributions. BMJ Glob Heal. 2022;7(6):e009592. Available from: https://pmc.ncbi.nlm.nih.gov/articles/PMC9161058/. Cited 2025 Jul 8. 10.1136/bmjgh-2022-009592 PMC9161058 35649633

[23] PRISMA for Scoping Reviews. Available from: http://www.prisma-statement.org/Extensions/ScopingReviews. Cited 2024 Jan 28.

[24] EMPHNET. Field Epidemiology Training Programs. Available from: https://emphnet.net/en/our-work/center-of-excellence-for-applied-epidemiology/field-epidemiology-training-programs/. Cited 2023 Apr 18.

[25] Gatei W, Galgalo T, Abade A, Henderson A, Rayfield M, McAlister D, et al. Field epidemiology and laboratory training program, where is the L-Track? Front Public Heal. 2018;6(SEP):1–10. 10.3389/fpubh.2018.00264 PMC6156269 30283768

[26] Team D, Dicker RC, Traicoff D, Gathany N, Davidson L, Evering-Watley M, et al. FETP-Frontline Planning Guide. 2020.

[27] Intermediate Field Epidemiology Training Program. Available from: https://emphnet.net/media/53xpaigs/40_2021_fetp-intermediate_project-brief.pdf. Cited 2023 Sep 5.

[28] André AMK, Lopez A, Perkins S, Lambert S, Chace L, Noudeke N, et al. Frontline Field Epidemiology Training Programs as a Strategy to Improve Disease Surveillance and Response. Emerg Infect Dis. 2017;23(Suppl 1):S166 Available from: /pmc/articles/PMC5711307/. Cited 2022 Nov 14. 29155657 10.3201/eid2313.170803 PMC5711307

[29] Collins D, Diallo BI, Bah MB, Bah M, Standley CJ, Corvil S, et al. Evaluation of the first two frontline cohorts of the field epidemiology training program in Guinea, West Africa. Hum Resour Health. 2022;20(1):1–12. Available from: 10.1186/s12960-022-00729-w. 35549712 10.1186/s12960-022-00729-w PMC9097411

[30] MacHaria D, Jinnai Y, Hirai M, Galgalo T, Lowther SA, Ekechi CO, et al. Impact of Kenya’s frontline epidemiology training program on outbreak detection and surveillance reporting: a geographical assessment, 2014-2017. Health Security. 2021;19(3):243–53. 33970691 10.1089/hs.2020.0042

[31] Kebebew T, Takele T, Zeynu N, Muluneh A, Habtetsion M, Kezali J, et al. A comparative cross-sectional evaluation of the field epidemiology training program-frontline in Ethiopia. BMC Public Health. 2022;22(1):1–9. Available from: https://bmcpublichealth.biomedcentral.com/articles/10.1186/s12889-022-13326-2. Cited 2023 Sep 5. 35538530 10.1186/s12889-022-13326-2 PMC9086414

[32] André AMK, Lopez A, Perkins S, Lambert S, Chace L, Noudeke N, et al. Frontline field epidemiology training programs as a strategy to improve disease surveillance and response. Emerg Infect Dis. 2017;23(Suppl 1):S166 Available from: /pmc/articles/PMC5711307/. Cited 2023 Sep 5. 29155657 10.3201/eid2313.170803 PMC5711307

[33] Ropa B, Flint J, O’Reilly M, Pavlin BI, Dagina R, Peni B, et al. Lessons from the first 6 years of an intervention-based field epidemiology training programme in Papua New Guinea, 2013–2018. BMJ Glob Heal. 2019;4(6):1–7. 10.1136/bmjgh-2019-001969 PMC6936504 31908873

[34] Training programs in Epidemiology and public health interventions network (TEPHINET). Germany Field Epidemiology Training Program (Postgraduate Training in Applied Epidemiology) | TEPHINET. Available from: https://www.tephinet.org/training-programs/germany-field-epidemiology-training-program-postgraduate-training-in-applied. Cited 2024 Apr 17.

[35] Subramanian RE, Herrera DG, Kelly PM. An evaluation of the global network of field epidemiology and laboratory training programmes: a resource for improving public health capacity and increasing the number of public health professionals worldwide. Hum Resour Health. 2013;11(1):1–7. 24053689 10.1186/1478-4491-11-45 PMC3849587

[36] Reddy C, Kuonza L, Ngobeni H, Mayet NT, Doyle TJ, Williams S. South Africa field epidemiology training program: developing and building applied epidemiology capacity, 2007–2016. BMC Public Health. 2019;19(3):1–8. Available from: https://bmcpublichealth.biomedcentral.com/articles/10.1186/s12889-019-6788-z. Cited 2023 Sep 5. 32326914 10.1186/s12889-019-6788-z PMC6696662

[37] Ragan P, Rowan A, Schulte J, Wiersma S. Florida epidemic intelligence service program: the first five years, 2001–2006. Public Health Rep. 2008;123(SUPPL. 1):21–7. 18497015 10.1177/00333549081230S108 PMC2233739

[38] Singh P, Gupta A, Tripathi A, Dhuria M, Aggarwal P. Developing public health capacities of frontline public health workforce in Uttarakhand. Indian J Community Heal. 2022;34(3):448–50. Available from: https://www.iapsmupuk.org/journal/index.php/IJCH/article/view/2476/1322. Cited 2023 Sep 5.

[39] Kumar R, Kateule E, Sinyange N, Malambo W, Kayeye S, Chizema E, et al. Zambia field epidemiology training program: Strengthening health security through workforce development. Pan Afr Med J. 2020;36(323):1–11. 33193977 10.11604/pamj.2020.36.323.20917 PMC7603807

[40] Al Nsour M, Iblan I, Tarawneh MR. Jordan field epidemiology training program: critical role in national and regional capacity building. JMIR Med Educ. 2018;20(4):1–7. 10.2196/mededu.9516 PMC5917079 29643050

[41] Tshimanga M, Gombe N, Shambira G, Nqobile N. Strengthening field epidemiology in Africa: the Zimbabwe field epidemiology training program. Pan Afr Med J. 2011;10(Suppl 1):12 Available from: /pmc/articles/PMC3266669/. Cited 2023 Sep 5. 22359700 PMC3266669

[42] Parry AE, Law C, Pourmarzi D, Vogt F, Field E, Colquhoun S. Contribution of the Australian field epidemiology training workforce to the COVID-19 response, 2020. West Pacific Surveill Response J WPSAR. 2022;13(4):1 Available from: /pmc/articles/PMC9912282/. Cited 2023 Sep 5. 36817505 10.5365/wpsar.2022.13.4.979 PMC9912282

[43] Ario AR, Bulage L, Kadobera D, Kwesiga B, Kabwama SN, Tusiime P, et al. Uganda public health fellowship program’s contribution to building a resilient and sustainable public health system in Uganda. Glob Health Action. 2019;12(1):1609825. Available from: 10.1080/16549716.2019.1609825. 31117889 10.1080/16549716.2019.1609825 PMC6534252

[44] Zodpey S, Pandey A, Murhekar M, Sharma A. Landscaping capacity-building initiatives in epidemiology in India: bridging the demand–supply gap. WHO South-East Asia J Public Health. 2015;4(2):204. 28607320 10.4103/2224-3151.206691

[45] Krause G, Aavitsland P, Alpers K, Barrasa A, Bremer V, Helynck B, et al. Differences and commonalities of national field epidemiology training programmes in Europe. Euro Surveill. 2009;14(43):1–7. 19883560

[46] European Centre for Disease Prevention and Control. Fellowship programme: EPIET/EUPHEM. 2022. Available from: https://www.ecdc.europa.eu/en/epiet-euphem. Cited 2022 Nov 14.

[47] López A, Cáceres VM. Central America Field Epidemiology Training Program (CA FETP): a pathway to sustainable public health capacity development. Hum Resour Health. 2008;6(1):1–6. 19087253 10.1186/1478-4491-6-27 PMC2636840

[48] Halm A, Seyler T, Mohamed S, Mbaé SBA, Randrianarivo-Solofoniaina AE, Ratsitorahina M, et al. Four years into the indian ocean field epidemiology training programme. Pan Afr Med J. 2017;26:1–11. 28674588 10.11604/pamj.2017.26.195.10358 PMC5483346

[49] Nsubuga P, Johnson K, Tetteh C, Oundo J, Weathers A, Vaughan J, et al. Field epidemiology and laboratory training programs in sub-saharan Africa from 2004 to 2010: Need, the process, and prospects. Pan Afr Med J. 2011;10. Available from: https://pubmed.ncbi.nlm.nih.gov/22187606/. Cited 2022 Nov 14. 10.4314/pamj.v10i0.72235 PMC3224071 22187606

[50] Lokossou VK, Sombie I, Ahanhanzo CD, Brito C, Antara SN, Nguku PM, et al. Strengthening applied epidemiology in west africa: progress, gaps, and advancing a regional strategy to improve health security. Health Secur. 2021;19(1):88–99. 33290155 10.1089/hs.2019.0133

[51] Kuonza L, Tint KS, Harris B, Nabukenya I. Public health systems strengthening in Africa: the role of South Africa field epidemiology and laboratory training programme. Pan Afr Med J. 2011;10 Suppl 1(Suppl 1):8 Available from: /pmc/articles/PMC3266682/. Cited 2023 Sep 5. PMC3266682 22359696

[52] Bandoh DA, Kenu E, Ameme DK, Sackey SO, Wurapa F, Afari EA. Sustainability of a field epidemiology and laboratory training programme: the ghanaian story. Pan Afr Med J. 2019;33:1–10. 10.11604/pamj.2019.33.68.16431 PMC6689840 31448030

[53] Njenga MK, Traicoff D, Tetteh C, Likimani S, Oundo J, Breiman R, et al. Laboratory Epidemiologist : Skilled Partner in Field Epidemiology and Disease Surveillance in Kenya Published by : Palgrave Macmillan Journals Linked references are available on JSTOR for this article : Laboratory Epidemiologist : Skilled Partner in Field. 2008.

[54] Nguku P, Oyemakinde A, Sabitu K, Olayinka A, Ajayi I, Fawole O, et al. Training and service in public health, Nigeria Field Epidemiology and Laboratory Training, 2008–2014. Pan Afr Med J. 2014;18(Supp 1):2. 25328621 10.11694/pamj.supp.2014.18.1.4930 PMC4199351

[55] Monday B, Gitta SN, Wasswa P, Namusisi O, Bingi A, Musenero M, et al. Paradigm shift: contribution of field epidemiology training in advancing the “One Health” approach to strengthen disease surveillance and outbreak investigations in Africa. Pan Afr Med J. 2011;10 Suppl 1(Suppl 1):13 Available from: /pmc/articles/PMC3266677/. Cited 2023 Sep 5. PMC3266677 22359701

[56] Nyarko KM, Miller LA, Baughman AL, Katjiuanjo P, Evering-Watley M, Antara S, et al. The role of Namibia field epidemiology and laboratory training programme in strengthening the public health workforce in Namibia, 2012–2019. BMJ Glob Heal. 2021;6(4):2012–9. 10.1136/bmjgh-2021-005597 PMC8051409 33849899

[57] Training programs in Epidemiology and Public Health Interventions Network (TEPHINET). Regional Field Epidemiology Training Program for Veterinarians (Southeast Asia). Available from: https://www.tephinet.org/training-programs/regional-field-epidemiology-training-program-for-veterinarians-southeast-asia. Cited 2022 Nov 15.

[58] EMPHNET. Bangladesh FETP-V. Available from: https://emphnet.net/en/our-work/the-center-of-excellence-for-applied-epidemiology/field-epidemiology-training-programs/fetps-of-the-eastern-mediterranean/bangladesh-fetp-v/. Cited 2022 Nov 15.

[59] Stehling-Ariza T, Lefevre A, Calles D, Djawe K, Garfield R, Gerber M, et al. Establishment of CDC global rapid response team to ensure global health security. Emerg Infect Dis. 2017;23(December):S203–9. 29155672 10.3201/eid2313.170711 PMC5711298

[60] Lior L, Njoo H. Ready to Go! Canada’s new rapid response team. Canada Commun Dis Rep. 2015;41(S6):9–13 Available from: /pmc/articles/PMC5868710/. Cited 2023 Dec 19. 10.14745/ccdr.v41is6a02 PMC5868710 29769972

[61] Raftery P, Hossain M, Palmer J. An innovative and integrated model for global outbreak response and research - a case study of the UK Public Health Rapid Support Team (UK-PHRST). BMC Public Health. 2021;21(1):1–17. 34247621 10.1186/s12889-021-11433-0 PMC8273030

[62] Araj R, Odatallah A, Mofleh J, Samy S, Alaya NB, Alqasrawi S. Rapid response teams’ initiative: Critical role and impact on national and eastern Mediterranean regional emergency management capacity building. JMIR Public Health and Surveillance. 2019;5(4):e14349 Available from: /pmc/articles/PMC6822060/. Cited 2022 Nov 14. 31621636 10.2196/14349 PMC6822060

[63] Herpolsheimer J. ECOWAS and the Covid-19 Pandemic: Regional Responses and African Interregional Cooperation. 2020. Available from: www.recentglobe.uni-leipzig.de. Cited 2023 Dec 19.

[64] Vong S, Samuel R, Gould P, El Sakka H, Rana BJ, Pinyowiwat V, et al. Assessment of Ebola virus disease preparedness in the WHO South-East Asia Region. Bull World Health Organ. 2016;94(12):913–24. Available from: https://pubmed.ncbi.nlm.nih.gov/27994284/. Cited 2023 Dec 19. 27994284 10.2471/BLT.16.174441 PMC5153931

[65] Li A, Kasai T. The Asia Pacific Strategy for Emerging Diseases – a strategy for regional health security. West Pacific Surveill Response J WPSAR. 2011;2(1):6 Available from: /pmc/articles/PMC3729053/. Cited 2024 Mar 5. 10.5365/WPSAR.2011.2.1.001 PMC3729053 23908877

[66] Aceng JR, Ario AR, Muruta AN, Makumbi I, Nanyunja M, Komakech I, et al. Uganda’s experience in Ebola virus disease outbreak preparedness, 2018–2019. Global Health. 2020;16(1):1–12. Available from: https://globalizationandhealth.biomedcentral.com/articles/10.1186/s12992-020-00548-5. Cited 2023 Dec 19. 32192540 10.1186/s12992-020-00548-5 PMC7081536

[67] Owens MD, Rice J. The Angolan pandemic rapid response team: an assessment, improvement, and development analysis of the first self-sufficient African national response team curriculum. Disaster Med Public Health Prep. 2019;13(3):577–81. 30479245 10.1017/dmp.2018.122

[68] World Health Organization. Ebola Virus Disease - Consolidated Preparedness Checklist. 2015.

[69] Hamblion EL, Raftery P, Wendland A, Dweh E, Williams GS, George RNC, et al. The challenges of detecting and responding to a Lassa fever outbreak in an Ebola-affected setting. Int J Infect Dis. 2018;66:65–73. Available from: https://pubmed.ncbi.nlm.nih.gov/29138016/. Cited 2023 Dec 19. 29138016 10.1016/j.ijid.2017.11.007

[70] Leser KA, Hay MC, Henebry B, Virden J, Patel M, Luttrell-Freeman J, et al. An academic-health department community partnership to expand disease investigation and contact tracing capacity and efficiency during the COVID-19 pandemic. J Public Heal Manag Pract. 2021;28(1):16–22. 10.1097/PHH.0000000000001379 34016907

[71] Koetter P, Pelton M, Gonzalo J, Du P, Exten C, Bogale K, et al. Implementation and process of a COVID-19 contact tracing initiative: leveraging health professional students to extend the workforce during a pandemic. Am J Infect Control. 2020;48(12):1451–6 Available from: /pmc/articles/PMC7425552/. Cited 2022 Nov 14. 32798633 10.1016/j.ajic.2020.08.012 PMC7425552

[72] Westfall M, Forster M, Golston O, Taylor KD, White K, Reid MJA, et al. Real world feedback: how well did the virtual training academy prepare California’s COVID-19 contact tracing workforce? Front Public Heal. 2022;10(June):1–9. 10.3389/fpubh.2022.857674 PMC9273764 35836992

[73] Said D, Brinkwirth S, Taylor A, Markwart R, Eckmanns T. The containment scouts: first insights into an initiative to increase the public health workforce for contact tracing during the covid-19 pandemic in Germany. Int J Environ Res Public Health. 2021;18(17):9325. 34501912 10.3390/ijerph18179325 PMC8431212

[74] Al Serouri AA, Ghaleb YA, Al Aghbari LA, Al Amad MA, Alkohlani AS, Almoayed KA, et al. Field Epidemiology Training Program response to COVID-19 during a conflict: experience from Yemen. Front Public Health. 2021;9:1–8. 10.3389/fpubh.2021.688119 PMC8646099 34881214

[75] Harris JR, Kadobera D, Kwesiga B, Kabwama SN, Bulage L, Kyobe HB, et al. Improving the effectiveness of field epidemiology training programs: characteristics that facilitated effective response to the COVID-19 pandemic in Uganda. BMC Health Serv Res. 2022;22(1):1–12. Available from: https://bmchealthservres.biomedcentral.com/articles/10.1186/s12913-022-08781-x. Cited 2023 Sep 5. 36526999 10.1186/s12913-022-08781-x PMC9756722

[76] Bell E, Mittendorf C, Meyer E, Barnum O, Reddy C, Williams S, et al. Continuing contributions of field epidemiology training programs to global COVID-19 response. Emerg Infect Dis. 2022;28(Suppl 1):S129 Available from: /pmc/articles/PMC9745235/. Cited 2024 Apr 18. 36502386 10.3201/eid2813.220990 PMC9745235

[77] Singh SK, Dikid T, Dhuria M, Bahl A, Chandra R, Vaisakh TP, et al. India field epidemiology training program response to COVID-19 pandemic, 2020–2021. Emerg Infect Dis. 2022;28(13):S138-44 Available from: /pmc/articles/PMC9745222/. Cited 2023 Sep 5. 36502396 10.3201/eid2813.220563 PMC9745222

[78] Anantharam P, Hoffman A, Noonan M, Bugli D, Pechta L, Bornemann J, et al. Addressing Operational Challenges Faced by COVID-19 Public Health Rapid Response Teams in Non–United States Settings. Disaster Med Public Health Prep. 2021;1. Available from: https://www.cambridge.org/core/journals/disaster-medicine-and-public-health-preparedness/article/addressing-operational-challenges-faced-by-covid19-public-health-rapid-response-teams-in-nonunited-states-settings/5E668CC831BCF0428CEDAF04A20FAE72. Cited 2022 Dec 9. 10.1017/dmp.2020.487 PMC7985625 33719992

[79] Marsh C, Salmon S, Housen T, Flint J, Taylor J, Hapolo E, et al. Ready to respond: adapting rapid response team training in Papua New Guinea during the COVID-19 pandemic. West Pacific Surveill Response J. 2022;13(4):1 Available from: /pmc/articles/PMC9912294/. Cited 2023 Dec 19. 10.5365/wpsar.2022.13.4.981 PMC9912294 36817503

[80] Smidt A, Balandin S, Sigafoos J, Reed VA. The Kirkpatrick model: a useful tool for evaluating training outcomes. J Intellect Dev Disabil. 2009;34(3):266–74. 19681007 10.1080/13668250903093125

[81] Wilson K, Juya A, Abade A, Sembuche S, Leonard D, Harris J, et al. Evaluation of a new field epidemiology training program intermediate course to strengthen public health workforce capacity in Tanzania. Public Health Rep. 2021;136(5):575–83. 33541215 10.1177/0033354920974663 PMC8361556

[82] Laurent-Comlan M, Horváth C, Mittendorf C, Allan C, Turcios-Ruiz R. Democratic Republic of the Congo Field Epidemiology Training Program Advanced Level Evaluation Report. 2022. Available from: https://ajabuadvisors.com/wp-content/uploads/2022/02/DRC-FETP-Advanced-final-evaluation-report_ENGLISH_17-Feb-2022.pdf. Cited 2022 Nov 14.

[83] Lee MS, Kim EY, Lee SW. Experience of 16 years and its associated challenges in the field epidemiology training program in Korea. Epidemiol Health. 2017;39:e2017058. 29370686 10.4178/epih.e2017058 PMC5847970

[84] Cáceres VM, Sidibe S, Andre M, Traicoff D, Lambert S, King ME, et al. Surveillance training for ebola preparedness in Côte d’Ivoire, Guinea-Bissau, Senegal, and Mali. Emerg Infect Dis. 2017;23:S174–82. 29155654 10.3201/eid2313.170299 PMC5711303

[85] Bhatnagar T, Gupte MD, Hutin YJ, Kaur P, Kumaraswami V, Manickam P, et al. Seven years of the field epidemiology training programme (FETP) at Chennai, Tamil Nadu, India: an internal evaluation. Hum Resour Health. 2012;10:1–7. 23013473 10.1186/1478-4491-10-36 PMC3505457

[86] Sobelson RK, Young AC. Evaluation of a federally funded workforce development program: the Centers for Public Health Preparedness. Eval Program Plann. 2013;37:50–7. 23380597 10.1016/j.evalprogplan.2013.01.001 PMC5582970

[87] Dey P, Brown J, Sandars J, Young Y, Ruggles R, Bracebridge S. The United Kingdom field epidemiology training programme: meeting programme objectives. Euro Surveill. 2019;24(36):1–8. 10.2807/1560-7917.ES.2019.24.36.1900013 PMC6737827 31507267

[88] Samy S, Lami F, Rashak HA, Al Nsour M, Eid A, Khader YS, et al. Public health workers’ knowledge, attitude and practice regarding COVID-19: the impact of Field Epidemiology Training Program in the Eastern Mediterranean Region. J Public Health (Oxf). 2021;43:iii1-11. 34580723 10.1093/pubmed/fdab240 PMC8500047

[89] Al Nsour M, Khader Y, Al Serouri A, Bashier H, Osman S. Awareness and Preparedness of Field Epidemiology Training Program Graduates to Respond to COVID-19 in the Eastern Mediterranean Region: Cross-Sectional Study. JMIR Med Educ. 2020;6(1):e19047 Available from: /pmc/articles/PMC7505690/. Cited 2022 Nov 14. 32406852 10.2196/19047 PMC7505690

[90] Kandun IN, Samaan G, Santoso H, Kushadiwijaya H, Juwita R, Mohadir A, et al. Strengthening Indonesia’s field epidemiology training programme to address International Health Regulations requirements. Bull World Health Organ. 2010;88:211–5 Available from: /pmc/articles/PMC2828784/. Cited 2023 Sep 5. 20428389 10.2471/BLT.09.065367 PMC2828784

[91] Al Nsour M, Khader Y, Bashier H, Alsoukhni M. Evaluation of advanced field epidemiology training programs in the Eastern Mediterranean Region: a multi-country study. Front Public Health. 2021;9:1–8. 10.3389/fpubh.2021.684174 PMC8339192 34368057

[92] Lubogo M, Donewell B, Godbless L, Shabani S, Maeda J, Temba H, et al. Ebola virus disease outbreak; the role of field epidemiology training programme in the fight against the epidemic, Liberia, 2014. Pan Afr Med J. 2015;22(Supp 1):5. 26779298 10.11694/pamj.supp.2015.22.1.6053 PMC4709128

[93] Bensyl DM, King ME, Greiner A. Applied epidemiology training needs for the modern epidemiologist. Am J Epidemiol. 2019;188(5):830–5. Available from: https://pubmed.ncbi.nlm.nih.gov/30877297/. Cited 2022 Dec 3. 30877297 10.1093/aje/kwz052 PMC6608580

[94] Parry AE, Kirk MD, Durrheim DN, Olowokure B, Colquhoun SM, Housen T. Shaping applied epidemiology workforce training to strengthen emergency response: a global survey of applied epidemiologists, 2019–2020. Hum Resour Health. 2021;19(1):1–13. Available from: 10.1186/s12960-021-00603-1. 33926469 10.1186/s12960-021-00603-1 PMC8084259

[95] Parry AE, Kirk MD, Colquhoun S, Durrheim DN, Housen T. Leadership, politics, and communication: challenges of the epidemiology workforce during emergency response. Hum Resour Health. 2022;20(1):1–14. Available from: 10.1186/s12960-022-00727-y. 35410336 10.1186/s12960-022-00727-y PMC8995686

[96] Li Y, Hsu EB, Davis XM, Stennies GM, Pham NN, Fisher MC, et al. Public health emergency response leadership training: a qualitative assessment of existing educational opportunities and perceived facilitators, barriers, and priorities in professional development. J Public Health Manag Pract. 2022;28(1):E283–90. 33729200 10.1097/PHH.0000000000001321

[97] Al Serouri A, Jumaan A, Alkohlani A. Yemen field epidemiology training programme: a tool for strengthening the public health workforce. East Mediterr Health J. 2018;24(9):905–13. 30570123 10.26719/2018.24.9.905

[98] Greiner AL, Stehling-Ariza T, Bugli D, Hoffman A, Giese C, Moorhouse L, et al. Challenges in public health rapid response team management. Heal Secur. 2020;18(Suppl 1):S8 Available from: /pmc/articles/PMC8900190/. Cited 2022 Dec 7. 10.1089/hs.2019.0060 PMC8900190 32004121

[99] Shears P, Garavan C. The 2018/19 Ebola epidemic the Democratic Republic of the Congo (DRC): epidemiology, outbreak control, and conflict. Infect Prev Pract. 2020;2(1):100038 Available from: /pmc/articles/PMC8336035/. Cited 2024 Mar 6. 34368690 10.1016/j.infpip.2020.100038 PMC8336035

[100] Ferri M, Lloyd-Evans M. The contribution of veterinary public health to the management of the COVID-19 pandemic from a One Health perspective. One Heal. 2021;12:100230 Available from: /pmc/articles/PMC7912361/. Cited 2022 Dec 9. 10.1016/j.onehlt.2021.100230 PMC7912361 33681446

[101] Hossain AD, Jarolimova J, Elnaiem A, Huang CX, Richterman A, Ivers LC. Effectiveness of contact tracing in the control of infectious diseases: a systematic review. Lancet Public Heal. 2022;7(3):e259 Available from: /pmc/articles/PMC8847088/. Cited 2024 Jan 30. 10.1016/S2468-2667(22)00001-9 PMC8847088 35180434

[102] Pinto J, Dissanayake RB, Dhand N, Rojo-Gimeno C, Falzon LC, Akwar H, et al. Development of core competencies for field veterinary epidemiology training programs. Front Vet Sci. 2023;10:1143375 Available from: /pmc/articles/PMC10118009/. Cited 2024 Jan 30. 37089403 10.3389/fvets.2023.1143375 PMC10118009

[103] Food and Agriculture Organization of the United Nations. Developing field epidemiology training for veterinarians Technical guidelines and core competencies. 2021. Available from: 10.4060/cb7545en. Cited 2022 Dec 7.

[104] Saravanan KA, Panigrahi M, Kumar H, Rajawat D, Nayak SS, Bhushan B, et al. Role of genomics in combating COVID-19 pandemic. Gene. 2022;823:146387. 35248659 10.1016/j.gene.2022.146387 PMC8894692

[105] Colijn C, Jd Earn D, Dushoff J, Ogden NH, Li M, Knox N, et al. The need for linked genomic surveillance of SARS-CoV-2. Can Commun Dis Rep. 2022;48(4):131–9. Available from: 10.14745/ccdr.v48i04a03. Cited 2022 Dec 9. 35480703 10.14745/ccdr.v48i04a03 PMC9017802

[106] World Health Organization. Global genomic surveillance strategy 2022–2032. 2021. Available from: https://www.who.int/publications/i/item/9789240046979. Cited 2022 Dec 9. 10.2471/BLT.22.288220 PMC8958828 35386562

[107] The International Association of National Public Health Institutes. Home l IANPHI. Available from: https://www.ianphi.org/. Cited 2022 Nov 15.

[108] Global Field Epidemiology Partnership. Global Field Epidemiology Partnership. Available from: Cited 2025 Jun 24. https://gfep.info/

[109] The World Bank. Financial Intermediary Funds -The Pandemic Fund. 2023. Available from: https://fiftrustee.worldbank.org/en/about/unit/dfi/fiftrustee/fund-detail/pppr. Cited 2024 Feb 4.

