# Supplementary material for: Public health emergency response through field epidemiology training and rapid response teams – a scoping review
Source: BMC Public Health. 2026 May 1;26:1453. doi: 10.1186/s12889-026-27361-w (PMC13137752; doi:10.1186/s12889-026-27361-w)
Supplement: Supplementary file 4 — Additional file 4: Evaluation results of Field Epidemiology Training Programs (FETPs): Evaluation of the effectiveness of FETPs that is presented in the studies. [file 12889_2026_27361_MOESM4_ESM.pdf]

Additional File 4. Evaluation results of Field Epidemiology Training Programs (FETPs)

**Type of training**	**Type of outcome evaluated**	**Result**
FETP in EMR(91)	1- Application of learned skills and competencies in workplace<br>2- Engagement in key activities	1- Program allowed participants to conduct most field epidemiology activities<br>2- Engagement in managing surveillance data, creating surveillance reports, and outbreak investigations
The frontline FETPs in Guinea(29)	1- Knowledge, skills, or competencies<br>2- Engagement in key activities	1- Improvements in the level of knowledge and increase in competency scores from before to after completion of the training<br>2- Positive changes have been reported in surveillance and response activities since the graduate's visit during their training
Tanzanian intermediate FETPs(81)	1- Knowledge, skills, or competencies<br>2- Engagement in key activities	1- Improvements in the level of knowledge and increase in competency scores from before to after completion of the training<br>2- 52 out of 53 trainees completed an evaluation of a district-level surveillance system and 39 trainees engaged in real-time outbreak investigations and post outbreak preparedness
Korea FETP(83)	1- Knowledge, skills, or competencies	1- Improvements in the level of knowledge and increase in competency scores from before to after completion of the training
The Kenyan frontline FETP (30)	1- Application of learned skills and competencies in workplace	1- Participants reported that knowledge and skills learned in practices positively affecting worksites
FETP India.(85)	1- Knowledge, skills and competencies<br>2- Course rating, satisfaction or meeting expectations<br>3- Engagement in key activities<br>4- Impact on career or education level	1- Most graduates self-assessed themselves as proficient for all core competencies at the end of the program<br>2- Quantity as rather short or fair, while fieldwork duration was evaluated as right among the majority of participants<br>3- The program resulted in 106 outbreaks investigations from 2001-2007<br>4- Beneficial toward future career developments

Democratic Republic of the Congo advanced FETP(82)	1- Knowledge, skill, and competencies<br>2- Application of learned skills and competencies in workplace<br>3- Engagement in key activities<br>4- Impact on career/education level	1- Improvements in the level of knowledge and increase in competency scores from before to after completion<br>2- Participants reported that knowledge and skills learned in practices positively affecting worksites<br>3- Outbreak investigation, data analysis, and epidemiologic report writing has been reported<br>4- Beneficial toward future career developments
FETP in UK. (87)	1- Knowledge, skills and competencies<br>2- Application of skills to workplace<br>3- Impact on career or education level	1- The program contributed to the development of a skilled workforce in field epidemiology and increasing stakeholders' confidence.<br>2- Participants reported that knowledge and skills learned in practices positively affecting worksites<br>3- Beneficial toward future career developments
Public health workers' knowledge, attitude and practice regarding COVID-19: the impact of Field Epidemiology Training Program in the Eastern Mediterranean Region.(88)	1-Knowledge and skills	1- No significant difference was found between mean knowledge scores of FETP-trained compared with non-FETP-trained survey participants, but mean knowledge scores for FETP-trained were significantly higher for the effective preventive measures of COVID-19 at households and public places, and in attitude and practice regarding COVID-19 pandemic control.
Awareness and Preparedness of Field Epidemiology Training Program Graduates to Respond to COVID-19 in the Eastern Mediterranean Region: Cross-Sectional Study. (89)	1-Knowledge and skills	1- FETP graduates found to be aware of the main symptoms of COVID-19, mode of transmission, high-risk groups, and how to use personal protective equipment.
A comparative cross-sectional evaluation of the Field Epidemiology Training Program-Frontline in Ethiopia. (31)	1- Knowledge, skills<br>2-Engagment in key activities	1- Knowledge and skills improved among the trained group after the training compared to their status before enrollment.<br>2- Improved surveillance officers' perceived skills and knowledge, and the availability and quality of surveillance formats and reports among the

		trained group. The utilization of computer programs, specifically Microsoft Word, Excel, and PowerPoint for surveillance activities, was significantly better among the trained group.
Surveillance training for ebola preparedness in Côte d'Ivoire, Guinea-Bissau, Senegal, and Mali(84)	1- Knowledge, skills and competencies<br>2- Engagement in key activities	1- Significant improvements in knowledge and skills assessed using pretest and posttest trainings evaluations<br>2- 84% of participants mentioned that they conducted surveillance activities as part of their routine work when they began training
An evaluation of the global network of field epidemiology and laboratory training programmes: a resource for improving public health capacity and increasing the number of public health professionals worldwide(35)	1-Engagement in key activities	1- 4663 Investigations of acute health events, 2534 Oral or poster presentations delivered, 1255 Surveillance systems established or evaluated, 1001 Peer-reviewed articles published.

Abbreviation: FETPs, Field Epidemiology Training Programs
